# Photosynthetic acclimation of terrestrial and submerged leaves in the amphibious plant *Hygrophila difformis*

**DOI:** 10.1093/aobpla/plz009

**Published:** 2019-02-27

**Authors:** Genki Horiguchi, Kyosuke Nemoto, Tomomi Yokoyama, Naoki Hirotsu

**Affiliations:** 1 Graduate School of Life Sciences, Toyo University, Itakura-machi, Oura-gun, Gunma, Japan; 2 Faculty of Life Sciences, Toyo University, Itakura-machi, Oura-gun, Gunma, Japan

**Keywords:** Amphibious plant, bicarbonate ion transport, carbon concentrating mechanism (CCM), *Hygrophila difformis*, underwater photosynthesis

## Abstract

*Hygrophila difformis*, a heterophyllous amphibious plant, develops serrated or dissected leaves when grown in terrestrial or submerged conditions, respectively. In this study, we tested whether submerged leaves and ethylene-induced leaves of the heterophyllous, amphibious plant *H. difformis* have improved photosynthetic ability under submerged conditions. Also, we investigated how this amphibious plant photosynthesizes underwater and whether a HCO_3_^−^ transport system is present. We have analysed leaf morphology, measured underwater photosynthetic rates and HCO_3_^−^ affinity in *H. difformis* to determine if there are differences in acclimation ability dependent on growth conditions: terrestrial, submerged, terrestrial treated with ethylene and submerged treated with an ethylene inhibitor. Moreover, we measured time courses for changes in leaf anatomical characteristics and underwater photosynthesis in terrestrial leaves after submersion. Compared with the leaves of terrestrially grown plants, leaf thickness of submerged plants was significantly thinner. The stomatal density on the abaxial surface of submerged leaves was also reduced, and submerged plants had a significantly higher O_2_ evolution rate. When the leaves of terrestrially grown plants were treated with ethylene, their leaf morphology and underwater photosynthesis increased to levels comparable to those of submerged leaves. Underwater photosynthesis of terrestrial leaves was significantly higher by 5 days after submersion. In contrast, leaf morphology did not change after submergence. Submerged leaves and submerged terrestrial leaves were able to use bicarbonate but submerged terrestrial leaves had an intermediate ability to use HCO_3_^−^ that was between terrestrial leaves and submerged leaves. Ethoxyzolamide, an inhibitor of intracellular carbonic anhydrase, significantly inhibited underwater photosynthesis in submerged leaves. This amphibious plant acclimates to the submerged condition by changing leaf morphology and inducing a HCO_3_^−^ utilizing system, two processes that are regulated by ethylene.

## Teaser

Angiosperms invading water from air will have faced problems of carbon supply. How does an amphibious plant grow in both terrestrial and submerged conditions? This manuscript reports on the plasticity of morphological and physiological responses to submergence in an amphibious plant, *Hygrophila difformis*. This amphibious plant photosynthesizes underwater by utilizing HCO_3_^−^ and this ability to utilize HCO_3_^−^ is switched on when it is submerged. Ethylene is involved in this submergence acclimation by changing leaf morphology and inducing HCO_3_^−^ utilization.

## Introduction

Generally, higher land plants do not grow under submerged conditions and aquatic plants, such as *Egeria densa*, cannot grow in terrestrial growth conditions. On the other hand, amphibious plants can acclimate to both terrestrial and submerged conditions. Amphibious plants have been hypothesized to develop leaves optimized for each environment (Rascio *et al.* 1999; Mommer *et al.* 2005; Colmer *et al.* 2011; Klančnik *et al.* 2012). *Hygrophila difformis*, a heterophyllous amphibious plant, develops serrated and dissected leaves when grown in terrestrial and submerged conditions, respectively. Furthermore, dissected leaves develop when *H. difformis* is treated with ethylene under terrestrial growth conditions (Li *et al.* 2017). Although this morphological change is regulated by ethylene, it remains unknown whether ethylene induces the leaves to functionally acclimate with the underwater condition.

Terrestrial angiosperms growing in water face problems with carbon limitation. In the submerged condition, gas diffusion resistance is 10^4^ times higher than in the terrestrial condition (Smith and Walker 1980; Maberly and Madsen 2002), and stomatal gas exchange for photosynthesis and respiration is restricted. Underwater photosynthesis decreases due to limited CO_2_ uptake. Moreover, total inorganic carbon, the substrate for photosynthesis, exists as CO_2_ in the terrestrial condition but dissolution of carbon in water provides not only CO_2_ but also bicarbonate (HCO_3_^−^) and carbonate (HCO_3_^2−^) ions. The relative proportions of dissolved inorganic carbon (DIC) constituents (CO_2_, HCO_3_^−^ and HCO_3_^2−^) depend on ionic strength, temperature and pH (Schwarzenbach and Meier 1958). Around neutral pH and ambient temperature conditions, CO_2_ and HCO_3_^−^ are the dominant forms; the acquisition and assimilation of HCO_3_^−^ is an important mechanism for some plants to acclimate to the submerged condition (Raven and Beardall 2015). The growth rates of aquatic plants are different depending on the DIC constituents in the environment (Hussner *et al.* 2016; Dülger and Hussner 2017).

Underwater, the CO_2_ concentrating mechanism (CCM) is important for enabling plants to photosynthesize under low CO_2_ conditions. About 50 % of submerged angiosperms can use HCO_3_^−^ for photosynthesis (Madsen and Sand-Jensen 1991). *Hydrilla verticillate* (Holaday and Bowes 1980; Spencer *et al.* 1996; Magnin *et al.* 1997), *Elodea canadensis* (Elzenga and Prins 1989) and *E. densa* (Browse *et al.* 1979; Casati *et al.* 2000) can induce C4-type photosynthesis under limiting CO_2_ conditions. An alternative CCM, crassulacean acid metabolism (CAM), can be induced in underwater conditions by some isoetid species such as *Lobelia dortmanna*, *Littorella uniflora* and *Isoetes australis* (Robe 1990; Madsen *et al.* 2002; Pedersen *et al.* 2011). In contrast, the amphibious plant *Eleocharis vivipala* was reported to change from C4-type photosynthesis when growing in terrestrial conditions to C3-type photosynthesis when submerged (Ueno *et al.* 1988; Ueno 2001).

Among the CCMs, the ability to utilize HCO_3_^−^ is the most common strategy among both marine and freshwater macrophytes (Maberly and Madsen 2002). Some *Potamogeton* species are known to utilize HCO_3_^−^ for photosynthesis when submerged (Prins *et al.* 1982). Cyanobacteria and marine diatoms photosynthesize using a carbonic anhydrase (CA) and a HCO_3_^−^ transporter. Some HCO_3_^−^ transporters have been isolated; namely, BicA and SbtA from cyanobacteria (Price *et al.* 2004; Price 2011) and SLC4 from marine diatoms (Nakajima *et al.* 2013). In seagrasses, three mechanisms for HCO_3_^−^ utilization have been reported depending on differences in sensitivity to acetazolamide (AZ), ethoxyzolamide (EZ) and Tris(hydroxymethyl)aminomethane (TRIS) (Beer *et al.* 2002; Rubio *et al.* 2017; Poschenrieder *et al.* 2018). First, apoplastic dehydration of HCO_3_^−^ catalysed by CA (Uku *et al.* 2005). Second, the catalysed apoplastic dehydration of HCO_3_^−^ to CO_2_ in acidic regions generated by the activity of H^+^-ATPases (Beer *et al.* 2002; Uku *et al.* 2005). Third, the direct uptake of HCO_3_^−^ by symport with H^+^ (Beer *et al.* 2002; Uku *et al.* 2005). Recently, inorganic carbon flux including protein localization, interaction and function has been well characterized for the CCM of *Chlamydomonas* (Mackinder *et al.* 2017; Mackinder 2018); however, the HCO_3_^−^ transporters have not been isolated from amphibious plants nor have there been any reports that amphibious plants can induce a HCO_3_^−^ transport system when submerged.

In this study, we tested whether submerged leaves and ethylene-induced leaves of the heterophyllous, amphibious plant *H. difformis* have improved photosynthetic ability under submerged conditions. Also, we investigated how this amphibious plant photosynthesizes underwater and whether a HCO_3_^−^ transport system is present.

## Materials and Methods

### Plant material and growth conditions

An amphibious plant (*H. difformis*) was purchased at a market (Charm Co. Ltd, Gunma, Japan). Three seedlings derived from cloned cuttings were planted in 7.5-cm-diameter pots containing soil. Twenty pots were placed in two identical glass tanks (W30 × D30 × H40 cm) at 25 °C, 8-h photoperiod and a photosynthetically photon flux density (PPFD) of 200 µmol m^−2^ s^−1^ at plant level during the photoperiod for 2 weeks. The water level was kept below the soil surface level. After 2 weeks, the two water tanks were separated into a terrestrial tank containing 10 pots and a submerged tank containing 10 pots. Plants in the submerged tank were 20 cm water depth after adding 30 L tap water. The two tanks were incubated at the same light and temperature conditions as described above for 2 months until leaves had developed under both growing conditions. The upper-most fully expanded leaves produced at terrestrial tank and at submerged tank were used as terrestrial leaves (Ter) and submerged leaves (Sub), respectively, were used for following analysis.

### Hormone and inhibitor treatments

Three seedlings were planted in same pots described above and each pot was placed in a glass jar (ϕ15 × H20 cm with a transparent lid). Plants growing in eight glass jars were incubated at the same conditions as above; 25 °C, 8-h photoperiod at a PPFD of 200 µmol m^−2^ s^−1^ for 2 weeks, then two glass jars were treated with ethylene or the ethylene inhibitor silver thiosulfate (STS). The ethylene gas concentration in each jar was maintained at 0.1 ppm by injecting ethylene using a syringe every other day. Submerged plants were treated with a 0.2 mM STS solution to inhibit ethylene perception. The solution was renewed every 2 weeks. The upper-most fully expanded leaves produced at terrestrial jar with ethylene and at submerged jar with STS were used as ethylene-treated leaves (+Ethylene) and ethylene-inhibited leaves (+STS), respectively.

### Morphological observations

Leaf images were scanned (GT-X820, Seiko-Epson, Nagano, Japan), and leaf area and perimeter were determined using ImageJ ver1.45s (National Institutes of Health, Bethesda, MD, USA). Leaf form complexity was estimated from the dissection index (DI), calculated as $(leaf\;perimeter)/\sqrt{leaf\;area}$ (Li *et al.* 2017). Plant segments were sectioned using a hand microtome (THK, Kenis, Osaka, Japan) and leaf thicknesses were measured by light microscopy (BX41, Olympus, Tokyo, Japan). Stomatal density on the abaxial surface was determined using same microscope. Four to five biological replicates were analysed.

### Photosynthetic measurements

Oxygen evolution was measured with a liquid-phase O_2_ electrode (OXYG-1, Hansatech, Norfork, UK) and chlorophyll (Chl) fluorescence was measured with a Chl fluorometer (Junior-PAM, Walz, Effeltrich, Germany). A fiber optics cable linked to the Junior-PAM was inserted through a plunger hole of the liquid phase chamber (DW1/AD, Hansatech). Four leaf segments (~6 mm long, projected area of ~150 mm^2^) were used for photosynthetic measurements in buffer (10 mM NaHCO_3_, 1.5 mM KCl and 1 mM NaCl) and four biological replicates were analysed. The temperature was maintained at 25 °C and irradiance was provided by an LED light source (KL 1600 LED, Schott, Mainz, UK). The light response curve of oxygen evolution was obtained for PPFD values between 0 and 820 μmol m^−2^ s^−1^. Leaf segments were kept in the darkness for 15 min before the measurements commenced, and then dark respiration and two fluorescence parameters (F_0_ and Fm) were measured. After O_2_ evolution had reached a steady-state rate, two fluorescence parameters (F and Fm′) were measured. The quantum efficiency of PSII (ΦPSII) was calculated as (Fm′ − F)/Fm′ (Genty *et al.* 1989) and relative electron transport rate through PSII (rETR) was estimated as the product of ΦPSII and PPFD of actinic light. Non-photochemical quenching (NPQ) was calculated as (Fm − Fm′)/Fm′. Excess energy defined as the absorbed light energy neither used for electron transport nor consumed as thermal dissipation was calculated as (F − F_0_′)/Fm′ (Demmig-Adams *et al.* 1996; Kato *et al.* 2003).

After photosynthetic measurements, the chlorophyll content was measured. Leaf disks were extracted in 1.0 mL *N*,*N*-dimethylformamide (DMF) in a 2.0 mL micro tube. After overnight storage of the samples at 4 °C in the dark, the absorbance of the extract was measured at two wavelengths (663.8 and 646.8 nm) using a UV spectrophotometer (UV-1800, Shimadzu, Japan). The value of non-specific absorption at 750 nm was measured and subtracted. Chlorophyll concentrations were calculated by the method of Wellburn (1994), and the content was expressed per mm^2^ of leaf area.

### Detection of H_2_O_2_ accumulation

Historical H_2_O_2_ accumulation in the submerged condition was detected by 3,3′-diaminobenzidine (DAB) staining. Detached leaves of terrestrial (Ter), submerged (Sub), ethylene-treated (+Et) and ethylene-inhibited (+STS) leaves with four biological replicates were placed in six-well microplates and submerged in the water with 0.5 mg mL^−1^ DAB and 0.01 % (v/v) Triton-X. Plates were placed in a desiccator connected to a water tap aspirator and vacuum infiltrated to −0.09 Pa for 30 min. Samples were incubated at 25 °C with illumination (200 μmol m^−2^ s^−1^) for 6 h. After incubation, leaves were decolorized overnight with a decolouring solution (ethanol:lactic acid:glycerol = 4:1:1) and then scanned. The areas of DAB-stained spots and leaf area were determined by ImageJ and the ratio of the spots area to leaf area was calculated as the H_2_O_2_ accumulation.

### Time-course analysis

The time courses for changes in leaf anatomical characteristics and underwater photosynthesis in terrestrial leaves were measured every 5 days until 20 days after submergence with four biological replicates. Underwater photosynthesis and leaf anatomical characteristics were determined by the same methods described above. A time course for changes in leaves grown in the terrestrial condition grown same condition as above was used as the control.

### Estimation of HCO_3_^−^ use

A pH drift experiment was performed to estimate HCO_3_^−^ utilization. The terrestrial (Ter), submerged (Sub) and 10 days after submergence of terrestrial-grown leaves (submerged terrestrial leaves; Sub-Ter) with 3–4 biological replicates were used. Whole leaves were incubated in a medium containing 10 mM NaHCO_3_, 1.5 mM KCl and 1 mM NaCl at 25 °C with illumination (200 μmol m^−2^ s^−1^) for 6 h after measuring leaf area. After incubation, the sample’s medium pH was measured with a pH meter (LAQUA F-51, Horiba, Kyoto, Japan). Medium pH without a leaf was measured as a blank. The ability for HCO_3_^−^ utilization was calculated as (pH sample − pH blank)/leaf area.

The photosynthetic rates of plants acclimated to different carbon constituents were measured at pH 6.3 and pH 8.3, where CO_2_ and HCO_3_^−^ were measured by oxygen electrode. The medium pH was adjusted to pH 6 or pH 8.3 by adding 1 M HCl or NaOH to induce CO_2_ or HCO_3_^−^ use, respectively.

The oxygen evolution rate in submerged leaves with an inhibitor of HCO_3_^−^ use was measured as described above with a PPFD of 285 μmol m^−2^ s^−1^. A measurement buffer containing 0.1 mM AZ, an inhibitor of apoplastic CA or EZ, an inhibitor of intracellular CA. To inhibit diffusive boundary layer (DBL) acidification, 50 mM TRIS was used.

### Data analysis

JMP (SAS Institute, USA) was used for statistical analyses. For multiple comparisons, we used a one-way ANOVA and Tukey’s HSD test as a *post hoc* test. Data were determined to be statistically significant differences as *P* < 0.05.

## Results

### Differences in leaf morphology and anatomical characteristics

**Figure 1.**
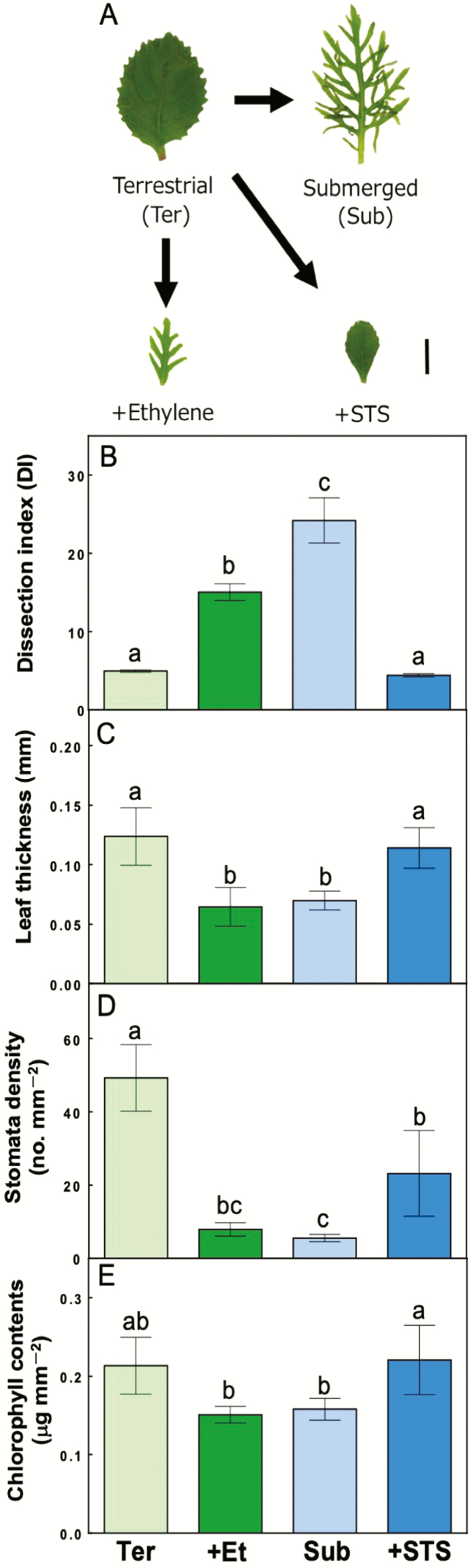
A comparison of leaf morphology and anatomical characteristics between different treatments. (A) Leaf morphology of *H. difformis* leaves grown under four different treatments: terrestrial (Ter), submerged (Sub), +Ethylene (terrestrial treated with 100 μL L^−1^ ethylene; +Et) and +STS (submerged treated with 0.2 mM STS). Scale bar = 10 mm. (B) Comparisons of the DI, (C) leaf thickness (mm), (D) stomatal density (number per mm^2^) and (E) chlorophyll content (μg mm^−2^) of leaves from plants grown under each treatment (means ± SD, *n* = 4–5). Light green, green, light blue and blue bars indicate terrestrial, +Ethylene, submerged and +STS treatments, respectively. Different letters indicate statistical differences between each type of treatment (Tukey’s HSD test, *P* < 0.05).

**Figure 2.**
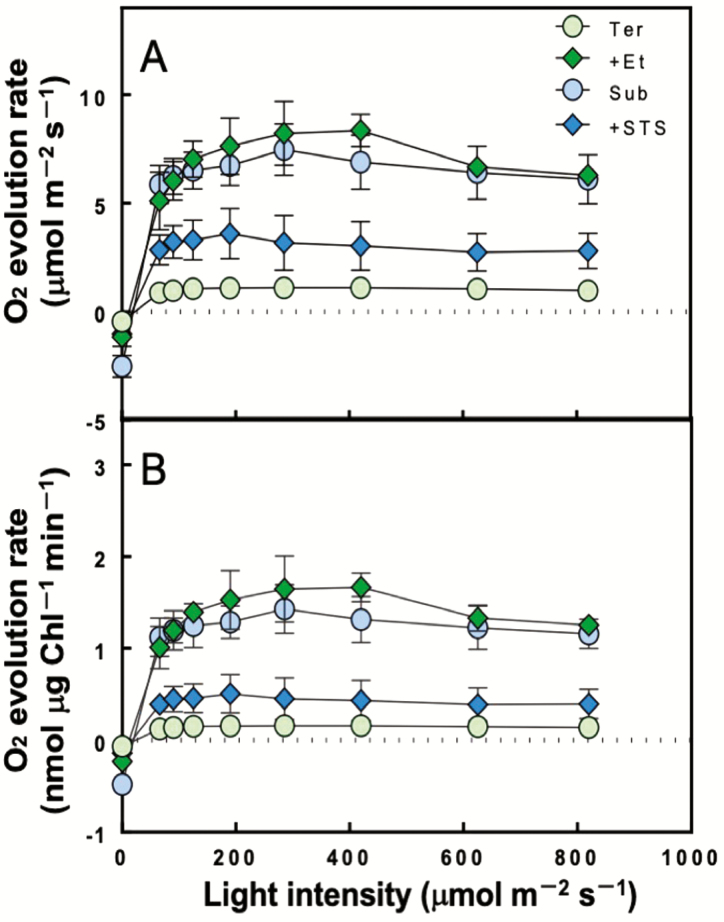
Underwater photosynthetic O_2_ evolution rates (μmol m^−2^ s^−1^) on a leaf area basis (A) and chlorophyll basis (B) with increasing light intensity in leaves that developed under each treatment (means ± SD, *n* = 4), terrestrial (Ter, green circles), submerged (Sub, blue circles), terrestrial treated with 100 μL L^−1^ ethylene (+Et, green diamonds) and submerged treated with 0.2 mM STS (+STS, blue diamonds). Measurements of the underwater conditions were made with an aqueous-phase O_2_ electrode at 25 °C. The measurement buffer consisted of 10 mM NaHCO_3_, 1.5 mM KCl and 1 mM NaCl.

Leaf morphology and anatomical characteristics were compared for each leaf treatment (Fig. 1). *Hygrophila difformis* formed leaves with a serrated edge when grown in the terrestrial condition and produced extremely dissected leaves when plants were submerged (Fig. 1A). The DI was larger in submerged leaves than in terrestrial leaves (Fig. 2B). Submerged leaves were significantly (*P* < 0.05) thinner and had a lower stomatal density on the abaxial surface compared with terrestrial leaves (Fig. 1C and D). There was no significant difference in the chlorophyll content between submerged and terrestrial leaves (Fig. 1E). Ethylene-treated *H. difformis* grown under terrestrial conditions formed dissected leaves that had an intermediate shape between terrestrial and submerged leaves (Fig. 1A and B). Leaves treated with ethylene had similar measurements of leaf thickness, stomatal density and chlorophyll content as submerged leaves, whereas these parameters were significantly lower than those from plants grown in the terrestrial condition (*P* < 0.05) (Fig. 1C–E). On the other hand, growth parameters of leaves formed under the submerged condition with an ethylene inhibitor (STS) were similar to those of leaves produced in the terrestrial condition (Fig. 1A and 1B). Silver thiosulfate treatment of plants did not change the leaf thickness or the chlorophyll content (Fig. 1C and E); however, stomatal density was reduced to an intermediate level between terrestrial and submerged leaves (Fig. 1D).

### Underwater photosynthesis

The light response of O_2_ evolution rates for each leaf segment was estimated on a leaf area basis (Fig. 2A) and chlorophyll basis (Fig. 2B). Whereas terrestrial leaves had low photosynthetic rates if submerged, submerged leaves attained high photosynthetic rates at all light intensities on both leaf area basis and chlorophyll basis (Fig. 2A and B). Underwater photosynthesis of ethylene-treated leaves rose to the same level as that of submerged leaves (Fig. 2A and B). In contrast, STS-treated leaves had a lower photosynthetic rate than submerged leaves and intermediate rate with terrestrial leaves.

**Figure 3.**
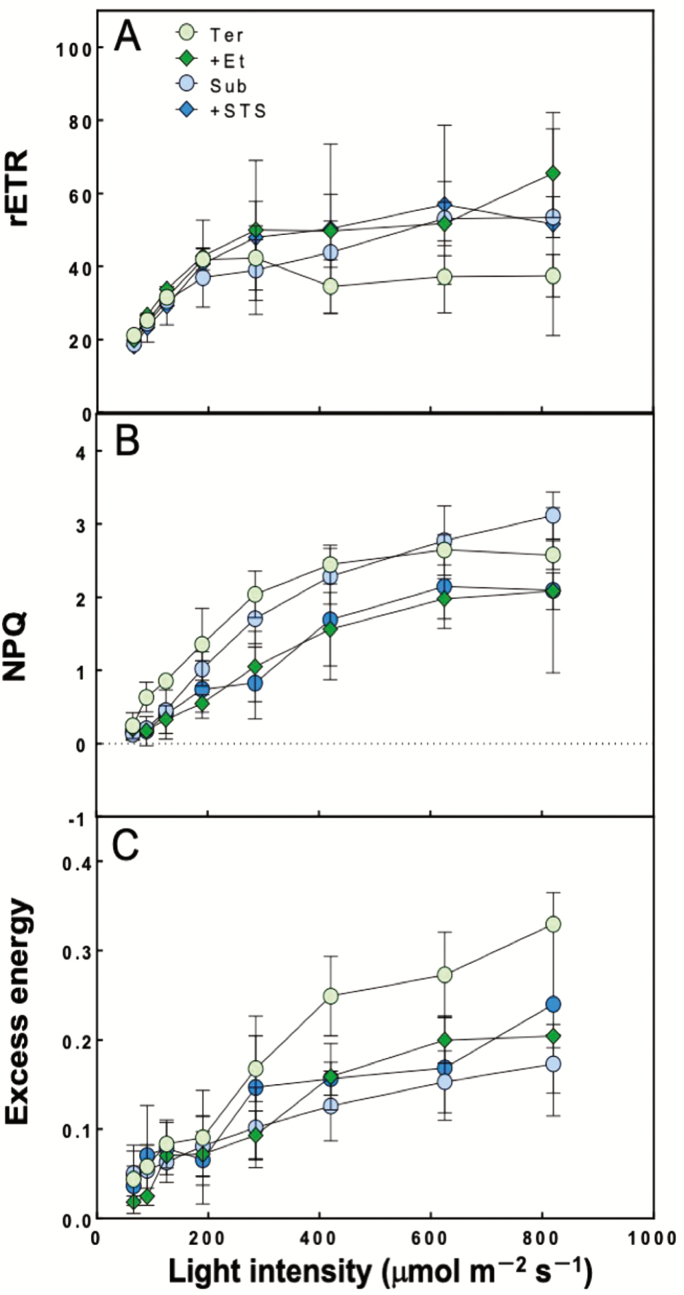
Response to light intensity of underwater chlorophyll fluorescence parameters in leaf segments from terrestrial (Ter, green circles), submerged (Sub, blue circles), terrestrial treated with 100 μL L^−1^ ethylene (+Et, green diamonds) and submerged treated with 0.2 mM STS (+STS, blue diamonds) (means ± SD, *n* = 4). (A) Relative ETR calculated by the product of ΦPSII and PPFD, (B) NPQ calculated as (Fm − Fm′)/Fm′, (C) the excess energy calculated as (F − F_0_′)/Fm′. Different letters indicate statistical differences between each leaf treatment (Tukey’s HSD test, *P* < 0.05).

Chlorophyll fluorescence parameters are shown in Fig. 3. There was no significant difference in the ETR and NPQ among the treatments (Fig. 3A and B). The excess energy in terrestrial leaves increased relative to the other treatments in light intensities above 420 μmol m^−2^ s^−1^ (Fig. 3C).

### Oxidative stress response under submerged conditions

**Figure 4.**
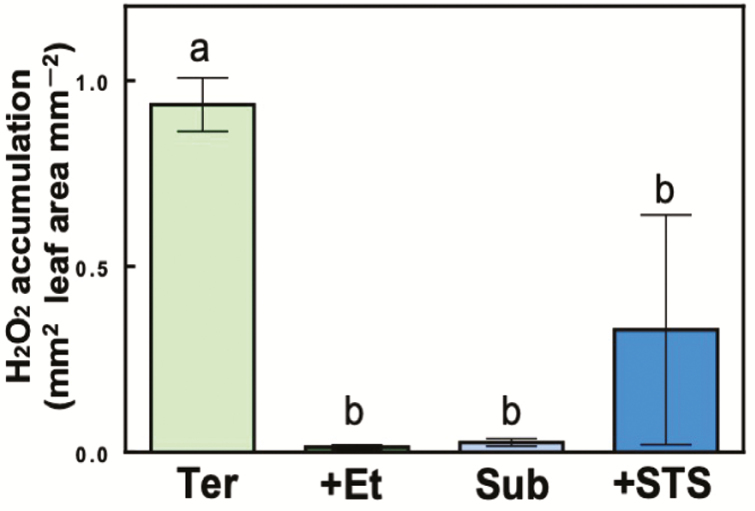
Detection of accumulated H_2_O_2_ in leaves using DAB staining. H_2_O_2_ accumulation of terrestrial leaves (Ter), submerged leaves (Sub), ethylene-treated terrestrial leaves (+Et) and ethylene-inhibited submerged leaves (+STS) after submergence with illumination for 6 h was calculated as the ratio of the area of DAB-stained spots to the leaf area (means ± SD, *n* = 4). Different letters indicate statistical differences between each leaf treatment (Tukey’s HSD test, *P* < 0.05).

Generally, plant causes oxidative stress when plants are exposed to light intensities that exceed their photosynthetic capacity. As shown in Fig. 4, H_2_O_2_ accumulated in terrestrial leaves when they were submerged and illuminated for 6 h, whereas almost no H_2_O_2_ accumulated in submerged or ethylene-treated terrestrial leaves.

### Time course of changes after submersion

**Figure 5.**
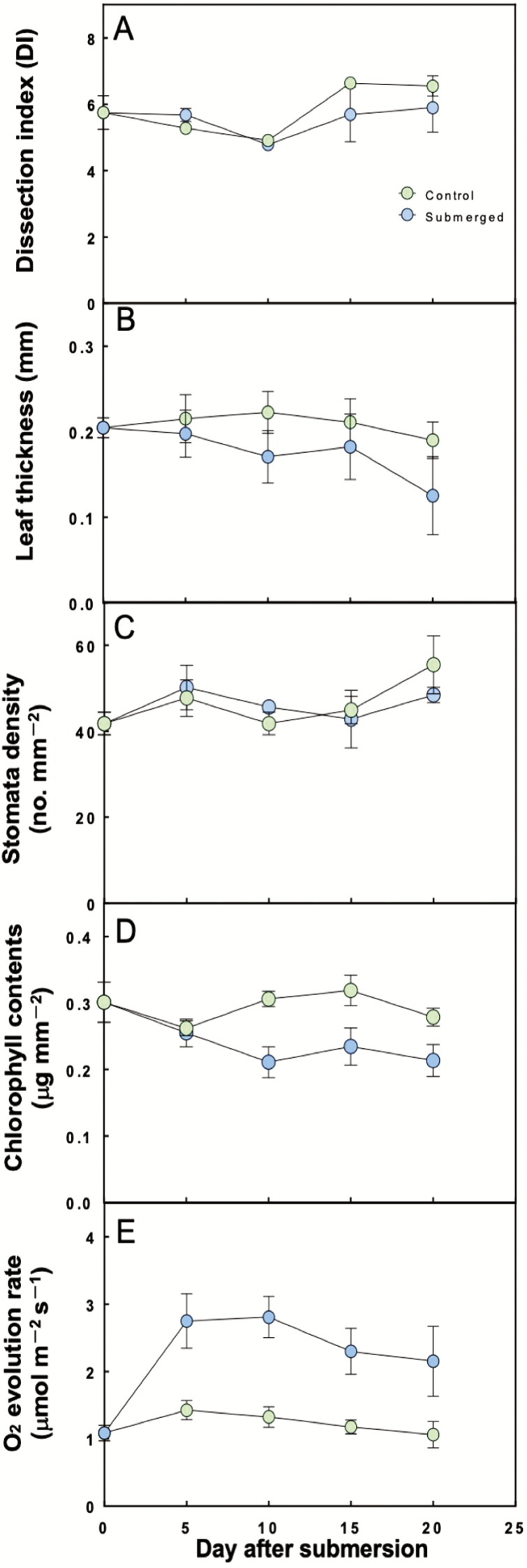
Time course of changes in underwater anatomical characteristics (A–D) and (E) photosynthesis (μmol O_2_ m^−2^ s^−1^) in the terrestrial leaves after submersion (means ± SD, *n* = 4). Blue circles (Submerged) are the terrestrial leaves after submergence. Green circles (Control) are the terrestrial-grown leaves as a control.

We evaluated the ability for terrestrially developed leaves to acclimate to changes in the environment by analysing how several parameters changed over time after submersion. Underwater photosynthesis significantly (*P* < 0.05) increased compared to the control at 5 days after submersion (Fig. 5E). Leaf thickness and chlorophyll contents were lower after submersion compared with the control (Fig. 5B and C). On the other hand, there were no significant differences in the DI and stomatal density after submersion compared with the control.

### HCO_3_^−^ use

**Figure 6.**
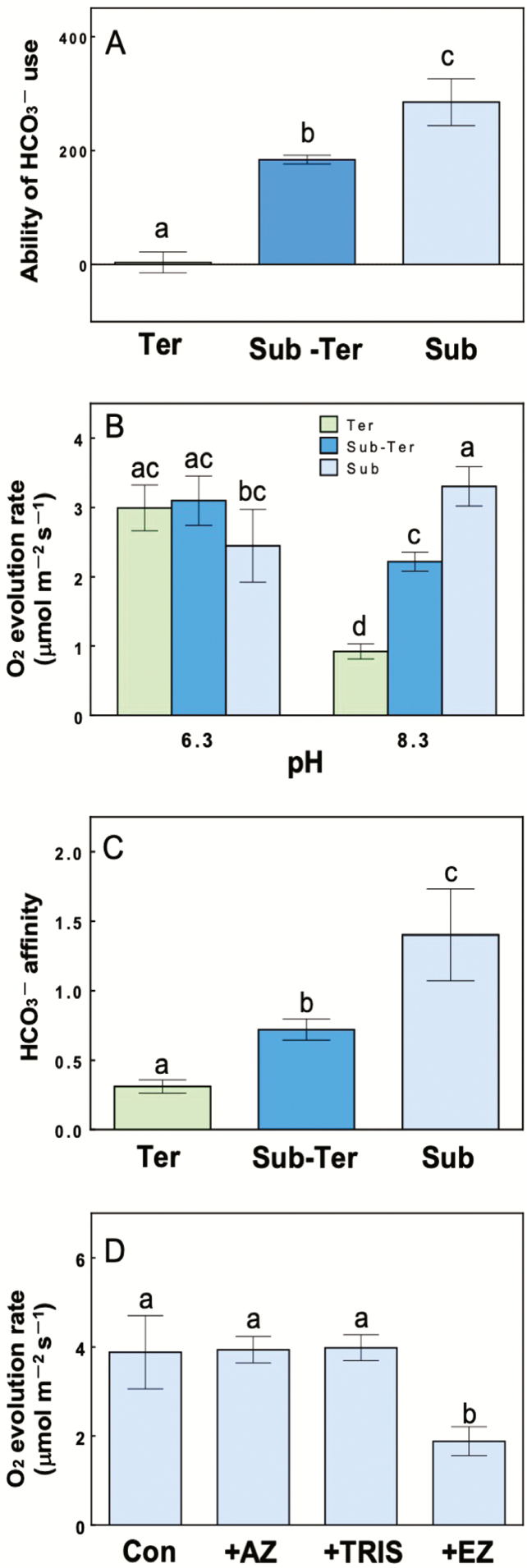
HCO_3_^−^ affinity in underwater photosynthesis of *H. difformis* (means ± SD, *n* = 3–4). Light green, blue and light blue bars indicate terrestrial (Ter), submerged-terrestrial (Sub-Ter) and submerged leaves (Sub), respectively. (A) Ability to use HCO_3_^−^ by leaves obtained during the pH drift experiments. Ability of HCO_3_^−^ use was calculated as (pH sample − pH blank)/leaf area, where pH sample is the pH in the medium after incubation with a leaf sample and pH blank is the pH in the medium leaf after incubation without a leaf sample. Whole leaves were incubated in a medium containing 10 mM NaHCO_3_, 1.5 mM KCl and 1 mM NaCl at 25 °C with illumination (200 μmol m^−2^ s^−1^) for 6 h. (B–D) The O_2_ evolution rate of leaf samples under different conditions. For each treatment, underwater photosynthesis was measured at 285 μmol m^−2^ s^−1^. (B) The O_2_ evolution rate by leaves submerged in a buffer at pH 8.3 or pH 6.3. (C) The ratio of underwater photosynthesis at pH 8.3/pH 6.3. (D) The O_2_ evolution rate of submerged leaves after exposure to measurement buffers containing 0.1 mM AZ, 0.1 mM EZ or 50 mM TRIS compared to the measurement buffer as a control (Con). Different letters indicate statistical differences between the treatments (Tukey’s HSD test, *P* < 0.05).

The ability to use HCO_3_^−^ was measured by a pH drift experiment. As an indication of HCO_3_^−^ use, the buffer pH of suspended leaf samples was measured and compared with the pH of a control that did not include a leaf sample (Maberly 1990). We measured the difference in HCO_3_^−^ utilization between treatments by the degree of increased pH per unit leaf area. Results for the pH drift experiment under illuminated conditions are shown in Fig. 6A. For terrestrial leaves, the pH did not change compared to the blank, whereas the pH of submerged leaves increased after illumination. The ability to use HCO_3_^−^ was greater for submerged leaves than for terrestrial leaves. Submerged terrestrial leaves had an intermediate ability to use HCO_3_^−^. When submerged leaves were incubated in the dark, a pH increase was not observed.

As shown in Fig. 6B, the photosynthetic rates for each type of leaf were measured at pH 6.3 and pH 8.3. The O_2_ evolution rate at pH 8.3 of submerged leaves was significantly higher than that of terrestrial leaves (*P* < 0.05). Submerged terrestrial leaves had an intermediate O_2_ evolution rate between terrestrial and submerged leaves. On the other hand, there were no significant differences in the O_2_ evolution rate at pH 6.3 between terrestrial leaves and submerged terrestrial leaves. The O_2_ evolution rate of terrestrial leaves and submerged terrestrial leaves was higher at pH 6.3 compared with measurements taken at pH 8.3. The affinity for HCO_3_^−^ in underwater photosynthesis is defined as the O_2_ evolution ratio at pH 8.3 relative to that at pH 6.3, and the HCO_3_^−^ affinity was higher in submerged leaves than terrestrial leaves (Fig. 6C). The HCO_3_^−^ affinity of submerged terrestrial leaves was intermediate between terrestrial and submerged leaves (Fig. 6C).

Ethoxyzolamide, an inhibitor of intracellular CA, significantly decreased the photosynthetic rate of submerged leaves (*P* < 0.05); however, the addition of AZ did not alter the rate of underwater photosynthesis in submerged leaves (Fig. 6D).

## Discussion

### Long-term acclimation to the submerged condition

Terrestrial leaves of *H. difformis* showed indications of oxidative stress when submerged and illuminated (Fig. 4). This oxidative stress seemed to be caused by a decreased photosynthetic rate at all light intensities (Fig. 2) and an increase in excess energy (Fig. 3D). On the other hand, submerged leaves did not shown signs of oxidative stress, indicating that *H. difformis* acclimated to the submerged condition by changing its leaf morphology (Fig. 1) and increasing its photosynthetic rate (Figs 2 and 3). Aquatic plants are usually characterized by their thin leaves without stomata, a reduced cuticle layer and a dissected or narrow shape (Kuwabara *et al.* 2003; Mommer *et al.* 2005; Klančnik *et al.* 2012; Kim *et al.* 2018). These characteristic are thought to enhance gas exchange from the leaf surface when submerged (Mommer *et al.* 2005; Colmer *et al.* 2011). That is, *H. difformis* acclimates to a submerged environment in the long term by developing leaves with a characteristic morphology that are capable of photosynthesis optimized for the submerged environment.

In previous studies, ethylene has been shown to play a role in the response to plant submersion. Ethylene regulates the promotion of internode elongation when plants are submerged, especially in deepwater rice (Kende *et al.* 1998; Jackson 2008). In heterophyllous, amphibious plants, such as *Callitriche heterophylla*, *Ludwigia arcuate* and *Ranunclus trichophyllus*, ethylene is induced to alter leaf morphology to that characteristic of submerged leaves (Deschamp and Cooke 1985; Kuwabara *et al.* 2001; Kim *et al.* 2018). After ethylene treatment of terrestrial plants in our study, leaf morphology and underwater photosynthetic ability were changed to the same levels as those of submerged leaves (Figs 1 and 2). Our results suggest that leaf morphology and photosynthetic function are regulated by ethylene to permit acclimation to the submerged condition.

### Acclimation to the submerged condition by terrestrial leaves

Underwater photosynthesis of terrestrial leaves was significantly higher by 5 days after submergence (Fig. 5E). In contrast, leaf morphology did not change after submergence, suggesting that *H. difformis* can acclimate to the submerged environment without changing leaf morphology. Although *H. difformis* can adapt from terrestrial to submerged conditions, the rate of photosynthesis in submerged terrestrial leaves was almost half that of submerged leaves (Figs 2 and 5E). These results suggest that this amphibious plant has the potential to adapt to submerged conditions even if its leaves develop under terrestrial conditions, and the adaptive ability varies depending on the conditions under which the leaves develop.

Results from the pH drift experiment and measurement of underwater photosynthesis at different pH values showed that submerged leaves and submerged terrestrial leaves were able to use bicarbonate but submerged terrestrial leaves had an intermediate ability to use HCO_3_^−^ that was between terrestrial leaves and submerged leaves (Fig. 6A and C). The ability to use HCO_3_^−^ seems to be inducible irrespective of the conditions in which the leaves developed or morphological changes. This finding shows the plant’s plasticity to safeguard against sudden submergence. On the other hand, the intensity of HCO_3_^−^ utilization depended on the conditions in which the leaves developed. Thus, the induction of the HCO_3_^−^ uptake mechanism might be regulated by stepwise factor(s) that are determined by the environment when the leaf developed.

### HCO_3_^−^ utilization for underwater photosynthesis

Terrestrial leaves had a low photosynthetic rate at pH 8.3, but underwater photosynthesis at pH 6.3 was higher (Fig. 6A), suggesting that the reason why terrestrial leaves do not photosynthesize in the submerged condition is that they cannot use HCO_3_^−^ for photosynthesis. In previous studies that used other freshwater plants, underwater photosynthesis had a higher level of DIC existing as CO_2_ than HCO_3_^−^ (Hussner *et al.* 2016; Dülger and Hussner 2017). Moreover, HCO_3_^−^ affinity was different among several growth conditions. That is, freshwater plants grown in a low CO_2_ condition had a higher affinity for HCO_3_^−^ compared to plants grown in a high CO_2_ condition (Hussner *et al.* 2016; Dülger and Hussner 2017). Marine diatoms grown in a high CO_2_ condition (5 % CO_2_) showed little net DIC uptake rate, whereas the net DIC uptake rate by low CO_2_-grown marine diatoms was as high as that in plants overexpressing SLC4, a Na^+^-dependent HCO_3_^−^ transporter (Nakajima *et al.* 2013). A positive correlation between relative growth rate (RGR) and HCO_3_^−^ affinity has been observed (Hussner *et al.* 2016), and HCO_3_^−^ affinity is considered an important factor in acclimating plants to submerged conditions. In our study, submerged leaves had a higher HCO_3_^−^ affinity than leaves produced in terrestrial conditions (Fig. 6C). The ratio of the photosynthetic rate at pH 8.3 relative to pH 6.3 of submerged leaves was above 1.0 (Fig. 6C), suggesting that submerged leaves can photosynthesize using HCO_3_^−^. We used three inhibitors of HCO_3_^−^ utilization (AZ, TRIS and EZ). Acetazolamide and TRIS can distinguish between the steps of proposed models for HCO_3_^−^ utilization (Beer *et al.* 2002; Hellblom and Axelsson 2003). Acetazolamide, a plasma membrane impermeable inhibitor, inhibits the catalysis of HCO_3_^−^ to CO_2_ by apoplastic CA. Tris(hydroxymethyl)aminomethane inhibits plasma membrane HCO_3_^−^/H^+^ symport by inhibiting the acidification of DBL following the apoplastic dehydration of HCO_3_^−^. Ethoxyzolamide is plasma membrane permeable and an inhibitor of intracellular CA, which is reported to inhibit the internal dehydration of HCO_3_^−^ by CA (Tortell *et al.* 1997; Rubio *et al.* 2017). Our study shows that underwater photosynthesis by submerged leaves was significantly inhibited by only the EZ treatment (*P* < 0.05), whereas TRIS had no effect. These results imply that submerged leaves of *H. difformis* can take up HCO_3_^−^ into the cell without involvement with H^+^. One possibility is decreased cuticle layer thickness in the submerged leaves (Mommer *et al.* 2005) allowed the diffusion of DIC into the cell. Another possibility is the involvement of HCO_3_^−^ transport not by any known HCO_3_^−^/H^+^ symport mechanism but other type transport like as Na^+^-dependent HCO_3_^−^ transporting system by SLC4 (Nakajima *et al.* 2013). Further detailed analysis to determine HCO_3_^−^ uptake mechanism in *H. difformis* will be required.

## Conclusion

*Hygrophila difformis* acclimates to a submerged environment by developing leaves with a characteristic morphology that are capable of photosynthesis optimized for the submerged environment. Ethylene treatment of terrestrial plants in our study, leaf morphology and underwater photosynthetic ability were changed to the same levels as those of submerged leaves, indicating that leaf morphology and photosynthetic function are regulated by ethylene to permit acclimation to the submerged condition. Furthermore, leaves acclimated to submerged condition had a high HCO_3_^−^ affinity. In this study, we showed that processes requiring CCM proteins such as HCO_3_^−^ transporters and CA are involved in photosynthetic acclimation by the amphibious plant *H. difformis*. This plant likely harbours genes and regulatory mechanisms that will prove to be valuable resources for discovering CCM genes in higher plants. Further characterization and understanding of this acclimation mechanism will provide novel resources for discovering CCM regulatory systems in higher plants.

## Sources of Funding

This work was supported by the INOUE ENRYO Memorial Grant, Toyo University.

## Contributions by the Authors

T.Y. and N.H. initially designed the research. G.H. and K.N. performed the experiment and G.H. and N.H. executed data interpretation. G.H. wrote the first draft of the manuscript and N.H. edited with input from all the authors.

## Conflict of Interest

None declared.
